# The effect of dehydration on muscle metabolism and time trial performance during prolonged cycling in males

**DOI:** 10.14814/phy2.12483

**Published:** 2015-08-21

**Authors:** Heather M Logan-Sprenger, George JF Heigenhauser, Graham L Jones, Lawrence L Spriet

**Affiliations:** 1 Canadian Sports Institute of OntarioToronto, Ontario, Canada; 2 Department of Medicine, McMaster UniversityHamilton, Ontario, Canada; 3 Department of Human Health and Nutritional Sciences, University of GuelphOntario, Canada

**Keywords:** Heat shock proteins, hydration, mild dehydration, muscle metabolism, performance

## Abstract

This study combined overnight fluid restriction with lack of fluid intake during prolonged cycling to determine the effects of dehydration on substrate oxidation, skeletal muscle metabolism, heat shock protein 72 (Hsp72) response, and time trial (TT) performance. Nine males cycled at ∼65% VO_2peak_ for 90 min followed by a TT (6 kJ/kg BM) either with fluid (HYD) or without fluid (DEH). Blood samples were taken every 20 min and muscle biopsies were taken at 0, 45, and 90 min of exercise and after the TT. DEH subjects started the trial with a −0.6% BM from overnight fluid restriction and were dehydrated by 1.4% after 45 min, 2.3% after 90 min of exercise, and 3.1% BM after the TT. There were no significant differences in oxygen uptake, carbon dioxide production, or total sweat loss between the trials. However, physiological parameters (heart rate [HR], rate of perceived exertion, core temperature [Tc], plasma osmolality [Posm], plasma volume [Pvol] loss, and Hsp72), and carbohydrate (CHO) oxidation and muscle glycogen use were greater during 90 min of moderate cycling when subjects progressed from 0.6% to 2.3% dehydration. TT performance was 13% slower when subjects began 2.3% and ended 3.1% dehydrated. Throughout the TT, Tc, Posm, blood and muscle lactate [La], and serum Hsp72 were higher, even while working at a lower power output (PO). The accelerated muscle glycogen use during 90 min of moderate intensity exercise with DEH did not affect subsequent TT performance, rather augmented Tc, RPE and the additional physiological factors were more important in slowing performance when dehydrated.

## Introduction

As little as 1–2% BM loss from sweating has been shown to compromise physiological functioning during prolonged exercise. Several studies have demonstrated that the magnitude of exercise-induced physiological responses such as core temperature (Tc), heart rate (HR), plasma osmolality (Posm), and central venous pressure (CVP) are exacerbated by dehydration and directly proportional to the degree of dehydration (Nadel 1980; Nadel et al. 1980; Montain and Coyle 1992; Armstrong et al. 1997; Sawka et al. 1985, 2007; Maughan and Meyer 2013). The reality in sport is that the majority of athletes only replace ∼50% of sweat losses during exercise leading to significant fluid deficits, and as a result experiences amplified physiological responses compared to when drinking enough fluid to replace sweat losses (Burke 1997; Krause and Rodrigues-Krause 2011; Logan-Sprenger et al. 2013a, 2013b).

Despite the large number of studies investigating the physiological responses to dehydration, very few have investigated the impact of dehydration on whole body substrate oxidation, skeletal muscle metabolism, and subsequent performance. One recent study conducted in recreationally active males who cycled for 2 h at 65% VO_2peak_ reported that muscle glycogen use was 24% greater in the dehydrated compared to the hydrated trial (Logan-Sprenger et al. 2013b). However, whole body CHO oxidation was not increased with dehydration to 2.7% BM loss. The relationship between the changes in substrate oxidation and muscle metabolism with progressive dehydration and performance was not examined in the previous study, so it is unknown if performance was affected. Dehydration has been shown to impair endurance performance. In a review by Cheuvront et al., the authors suggest that intravascular volume loss is likely one mechanism by which dehydration impairs aerobic performance (Cheuvront et al. 2010). However, what is not known is whether the performance detriments are a result of glycogen depletion or other physiological factors. Walsh et al. (Walsh et al. 1994) had trained males cycle for 60 min at 70% VO_2peak_ and then complete a time trial (TT) to exhaustion at 90% VO_2peak_ either consuming water with 120 mL NaCl every 10 min or no fluid. TT performance was significantly slower when subjects were dehydrated by ∼2% BM loss (fluid 9.8 ± 3.9 vs. fluid restricted 6.8 ± 3.0 min). However, in response to a similar hydration status (progressive loss to 2.7% BM loss), no significant differences were reported when trained men completed a 40 km cycling TT performance as measured by power output and mean finish time (Berkulo et al. 2015). Furthermore, when trained subjects are dehydrated to a greater extent pre-exercise (∼3% BM loss), impaired exercise capacity and significantly increased core temperature were reported in response to an incremental cycling to exhaustion protocol in the heat (35°C) (Trangmar et al. 2014). In these studies alterations in metabolism were not ascertained, so the contribution of metabolism to the performance detriments remains unclear.

Therefore, the purpose of this study was to further investigate the effects of progressive dehydration on TT performance and several metabolic, physiologic, and mental variables that may be related to performance in active males. By adopting an overnight fluid restriction protocol subjects were mildly dehydrated before starting the exercise protocol. They then continued to dehydrate during the moderate intensity exercise and following TT. We hypothesized that carbohydrate oxidation, muscle glycogen use, cellular stress (heat shock protein 72 response), and all other physiological measures (HR, Tc, plasma volume [Pvol] loss) would be greater with dehydration during moderate intensity exercise compared to when hydrated. As a result, we predicted that TT performance would be significantly slower. By measuring intermediate blood (every 20 min) and skeletal muscle measurements (0, 45, 90 min, and post-TT), mental responses (rating of perceived exertion [RPE]), and other physiological parameters (Tc, HR), the study attempted to identify the physiological factors that were contributing to performance detriments when dehydrated.

## Methods

### Subjects

Nine trained males, mean age 21.6 ± 0.5 years, height 178.1 ± 0.9 cm, weight 77.5 ± 3.0 kg, and VO_2peak_ 4.4 ± 0.2 L/min, participated in the study. All subjects engaged in physical activity 4–6 days/week. On average, subjects cycled for ∼4–5 h/week. Subjects were informed both verbally and in writing of the experimental protocol and potential risks prior to giving their written consent to participate. The Research Ethics Boards of the University of Guelph and McMaster University approved the study.

### Pre-experimental protocol

In preparation for the experiment, subjects visited the laboratory on three separate occasions. On the first visit, subjects performed an incremental cycling test to exhaustion on an electronically braked cycle ergometer (LODE Excalibur, Quinton Instrument, Groningen, the Netherlands) for the determination of VO_2peak_. Respiratory gases were collected and analyzed using a metabolic cart (MOXUS metabolic system, AEI Technologies, Pittsburgh, PA). Following a 30-min break, subjects cycled for ∼20 min at ∼65% VO_2peak_ to establish the power output for the subsequent 90-min trial.

On two subsequent occasions, subjects reported to the laboratory for practice trials and cycled at ∼65% VO_2peak_ for 90 min followed by a TT. For the first practice trial, subjects completed the trial without fluid (DEH) to ascertain sweat loss over the entire trial and determine how much fluid each participant needed to drink throughout the second practice trial (HYD) to maintain fluid balance.

Subjects woke, urinated, and took their body mass (SECA model 874 with precision to 0.01 kg) in shorts for five consecutive days prior to each trial to ascertain mean morning BM. All subjects abstained from strenuous exercise and caffeine, and recorded their diet in the 24 h before each trial. Subjects in the DEH trial abstained from drinking fluid from 6 pm the evening before the trial until they arrived at the laboratory. Two hours prior to the practice rides, subjects ingested a meal provided for them (790 kcal; 144 g carbohydrate, 35 g fat, 19 g protein) and 250 mL of fluid. Additionally, subjects in the HYD trial drank as they normally do the night before and drank 300 mL of water 90 and 45 min before the trial to ensure they were well hydrated before cycling. This morning protocol was replicated in its entirety for each practice and experimental trial. Upon arrival to the laboratory, subjects voided their bladder and provided a small midstream urine sample to determine urine specific gravity (USG) and urine osmolality (Uosm) and completely voided their bladder. Subjects were weighed in shorts to determine pretrial BM. The effect of the overnight fluid dehydration protocol was ascertained by subtracting the pretrial BM from the mean BM measurements of the five consecutive mornings prior to each trial. Following 45 and 90 min of exercise, subjects stopped cycling and dismounted the cycle ergometer, removed their shoes and shirt, toweled dry, and were weighed wearing only shorts for the determination of sweat loss during the previous 45 min of exercise. At 45 min, subjects were put on a dry t-shirt and recommenced cycling. Any urine produced was included in the calculation of sweat loss (Logan-Sprenger et al. 2011, 2013b).

Three-minute respiratory gas measurements were collected every 20 min during exercise to determine the volume of oxygen consumed (VO_2_), the volume of carbon dioxide produced (VCO_2_), and to calculate the respiratory exchange ratio (Peronnet and Massicotte 1991) and whole body carbohydrate (CHO) and fat oxidation with use of the nonprotein RER table and the following equations: *CHO oxidation* (g) = 4.585 (VCO_2_) − 3.226 (VO_2_), and *fat oxidation* (g) = 1.695 (VO_2_) − 1.701 (VCO_2_) (Saltin 1980; Ferrannini 1988). After each respiratory gas measurement, subjects completed a 2 min simulated hill climb at 85% VO_2peak_ for a total of four intervals to incur greater sweat loss over the 90-min trial.

Following the 90-min trial at ∼65% VO_2peak_, subjects were rested for 5–7 min. Subjects then remounted the cycle ergometer and completed a TT where they were asked to complete 6 kilojoules (kJ)/kg BM of work in the fastest time possible. The total work (kJ) that each subject needed to complete was calculated from the subject’s mean morning BM for that trial. Monetary prizes were established to motivate subjects to work hard and finish each TT in the fastest time. Subjects were reminded of the monetary prizes associated with the fastest cumulative TT over the four trials (practice + experimental) before commencing each trial. Throughout the trial, subjects were blinded to elapsed time and power output but were able to see the amount of work (kJ) completed. After the TT, subjects removed their shoes and shirt, toweled dry, and were weighed for the determination of BM loss over the TT. Practice trials were separated by 5–7 days.

### Experimental protocol

Subjects arrived to the laboratory on two occasions for the actual experiment. During the experimental trials, subjects cycled at ∼65% VO_2peak_ for 90 min followed by a TT where subjects completed 6 kJ/kg BM of work in the fastest time possible. In one trial, subjects drank fluid to match sweat losses (HYD) or were without fluid (DEH). Subjects replicated the same procedure as described earlier for the practice trials. In addition, heart rate (HR) was collected using a Polar RS400 downloadable HR monitor (Polar Electro., Lachine, QC) and core temperature (Tc) was determined using a calibrated ingestible thermistor (HQ Inc., Palmetto, FL) that was ingested 3–4 h before each trial. Prior to exercise, a Teflon catheter was inserted into an antecubital vein for blood sampling and was flushed with 0.9% saline to maintain patency. One leg was also prepared for percutaneuous needle biopsy sampling of the vastus lateralis muscle by the Bergström technique (Bergström 1962). Three incisions were made in the skin and deep fascia under local anesthesia (2% xylocaine without epinephrine) for three separate biopsies. Venous blood samples were obtained at 20, 40, 60, 75, and 90 min of exercise. HR, Tc, and RPE were recorded every 10 min during exercise (Borg 1970). During the HYD trial subjects were given fluid every 15 min to match sweat loss. At 45 and 90 min, the subject stopped cycling and a muscle biopsy was taken with the subject sitting on the cycle ergometer. During the TT, power output, HR, Tc, and RPMs were recorded at 15%, 30%, 45%, 60%, 75%, 90%, and 100% of total work (kJ) completed. Also, a venous blood sample was taken at 50% (TT1) and 90% (TT2) of total work completed. Immediately following the completion of the TT a muscle biopsy was taken with the subject sitting on the cycle ergometer. After the muscle biopsy was taken, subjects removed their shoes and shirt, toweled dry, and were weighed for the determination of BM loss over the TT. The same procedure was replicated for the second trial with muscle biopsies taken from the opposite leg. The trials were randomized and separated by 7 days.

### Analyses

#### Trial conditions

Laboratory temperature (°C) and relative humidity (%) were measured using a Digital Thermometer (Fisher Scientific, Ottawa, ON). USG was determined from the initial urine sample using a handheld pocket refractometer (ATAGO USA Inc., Bellevue, WA) to determine pretrial hydration status. The refractometer was calibrated with distilled water prior to its use and checked periodically between urine samples. USG values were classified as: USG < 1.020 signifying a hydrated state and a USG ≥ 1.020 indicating hypohydration (Casa et al. 2000; Sawka et al. 2007). Uosm was determined by freezing point depression (Gonotec Osmomat 030 Cryoscopic Osmometer; Gonotec, Berlin, Germany).

#### Blood measurements

Venous blood was collected in sodium heparin tubes. A portion of whole blood (200 *μ*L) was added to 1 mL of 0.6 mol/L perchloric acid and centrifuged. The supernatant was stored at −20°C and later analyzed for blood glucose and lactate with fluorometric techniques (Bergmeyer 1974). A second portion (1.5 mL) was centrifuged and the supernatant was analyzed for plasma free fatty acids (FFA) with an enzymatic colorimetric technique (NEFA C test kit, Wako Chemicals, Richmond, VA). A third portion (1.5 mL) was added to 30 mL of EGTA and reduced glutathione and centrifuged (10,000 *g*) for 3 min, and the supernatant was analyzed for epinephrine with an enzymatic immunoassay kit (Epinephrine RIA kit, Rocky Mountain Diagnostics Inc., Colorado Springs, CO). Venous blood was used for the determination of whole blood hemoglobin (Hb) and hematocrit (Hct). Hb was measured in duplicate using an automated blood analysis machine (OSM3 Hemoximeter; Radiometer, Copenhagen, Denmark). Hct was measured in triplicate using capillary tubes and a microhematocrit centrifuge and reader (Micro-capillary reader, Damon/IEC Division, USA). The percent plasma volume change (%Pvol) was calculated using whole blood Hb and Hct measurements (Dill and Costill 1974). The remaining venous blood was allowed to clot and the serum was separated by centrifugation at 3307 *g* for 10 min at 4°C. Serum was refrigerated before the analysis for osmolality by freezing point depression (Gonotec Osmomat 030 Cryoscopic Osmometer; Gonotec, Berlin, Germany).

#### Muscle metabolites

Each muscle biopsy was freeze dried, powdered, and dissected free of visible connective tissue, fat, and blood. One aliquot of freeze-dried powdered muscle (∼10 mg) was extracted in 0.5 mol/L HCLO_4_–1 mmol/L EDTA and neutralized with 2.2 mol/L KHCO_3_. The supernatant was used to measure phosphocreatine (PCr), creatine (Cr), adenosine triphosphate (ATP), and lactate (Bergmeyer 1974). Muscle metabolites were normalized to the highest total Cr content measured from all biopsies from each subject. Muscle glycogen content was determined in duplicate using two additional aliquots of freeze dried muscle (2–4 mg). Glycogen was extracted in 0.1 mol/L NaOH and neutralized with 0.1 mol/L HCL–0.2 mol/L citric acid–0.2 mol/L Na_2_PO_4_, and amyloglucosidase was added to degrade glycogen to glucose, which was measured spectrophotometrically and normalized for total Cr (Bergmeyer 1974).

#### Muscle calculations

Free ADP (ADPf) and AMP (AMPf) contents were calculated by assuming equilibrium of the creatine kinase and adenylate kinase reactions (Dudley et al. 1987). Specifically, ADPf was calculated using the measured ATP, Cr, and PCr values, an estimated H^+^ concentration, and the creatine kinase constant of 1.66 × 109 (Saltin 1980). AMPf was calculated from the estimated ADPf and measured ATP content using the adenylate kinase equilibrium constant of 1.05.

*Hsp72* western blots. Skeletal muscle tissue from the vastus lateralis was homogenized in ice-cold buffer (1:9 wt/vol dilution) suitable for whole cell protein extraction and preserving phosphorylation states of proteins. Homogenates were sonicated for 5 sec to ensure the nuclear membrane was completely broken, centrifuged at 1500 *g* for 15 min at 4°C, and the supernatant was removed, and protein content was determined using BSA as standards. Whole cell lysate protein was mixed with equal volumes of sample buffer (0.5 mol/L Tris base, 13% glycerol, 0.5% SDS, 13% *β*-mercaptoethanol, and bromophenol blue) and separated according to their molecular weight on gels consisting of a 12% acrylamide separating gel overlaid by a 4% acrylamide stacking gel. A molecular weight standard (catalog no. 161-0373 Bio-Rad) was run concurrently on each gel for accurate determination of the proper molecular weight of the protein. After electrophoresis, proteins were transferred to polyvinylidene difluoride membranes and blocked in Tris-buffered saline (TBS) for 1 h and then washed twice with 0.01% Tween 20 in TBS (TTBS) for 5 min each wash. Membranes were then incubated at 4°C in primary antibody specific to Hsp72 (anti-Hsp70 polyclonal antibody, 1:5000, SPA-812, Stressgen) in TTBS (2% BSA, catalog no. A-2153, Sigma). Following incubation, membranes were washed in TTBS and incubated with secondary antibody according to the manufacturer’s instructions. Antibody detection was performed using the enhanced chemiluminescence method (Syngene Chemigenius2; PerkinElmer, Waltham, MA), and quantified with densitometry (Gene Tools software; PerkinElmer). Repeats were done on all samples. Ponceau staining was performed after quantification to ensure loading was even in each well.

### Statistical analysis

All data were tested for normality of distribution and presented as the mean ± standard deviation (SD). Time versus trial data were assessed using a two-way ANOVA and specific differences were located using the Student–Newman–Keuls post hoc test. A paired *t*-test was used to compare single parameter data between trials. Statistical significance was accepted as *P *<* *0.05.

## Results

### Trial conditions

No pretrial differences existed between the HYD and DEH trials for laboratory temperature (HYD, 23 ± 0.1°C vs. DEH, 23 ± 0.2°C) and relative humidity (32 ± 0.3% vs. 33 ± 0.4%). Pretrial hydration status was significantly different between trials based on USG (HYD, 1.015 ± 0.002 vs. DEH, 1.024 ± 0.002) and Uosm (700 ± 111 mmol/L vs. 968 ± 49 mmol/L). Posm was not significantly different between trials (288 ± 1.5 mOsmol/kg^−1^ vs. 290 ± 1.1 mOsmol/kg^−1^) (Table2).

### Body mass loss, sweat loss, and fluid intake

In the HYD trial, subjects drank a mean of 1.6 ± 0.2 L of fluid and BM decreased by only 0.5 kg or 0.6% BM (Table1). As a result of overnight fluid restriction, DEH subjects started the trial with a BM loss of 0.6% (0.5 ± 0.1 kg). BM was lower at 45, 90 min, and after the TT in the DEH versus HYD trial and there was no difference in sweat loss between trials (Table1).

**Table 1:** Differences in sweat loss, body mass (BM), and percent (%) BM loss between the hydrated (HYD) and dehydrated (DEH) trial at 0, 45, and 90 min of cycling at ∼65% VO_2peak_ and after the time trial

Time (min)	HYD					DEH				
	Mean morning BM		0–45	45–90	90–Post TT	Mean morning BM		0–45	45–90	90–Post TT
Sweat			0.7 ± 0.1	0.8 ± 0.1	0.8 ± 0.1			0.8 ± 0.1	0.9 ± 0.1	0.7 ± 0.1
loss (L)										
		0	45	90	Post-TT		0	45	90	Post-TT
BM (kg)	77.3 ± 3.0	77.4 ± 2.9	77.2 ± 2.8	77.1 ± 2.8	76.8 ± 2.7	77.6 ± 3.0	77.1 ± 3.1	76.5 ± 3.1*	75.8 ± 3.1*	75.2 ± 3.1*
BM loss (%)	–	–	–	–	−0.6 ± 0.1	–	−0.6 ± 0.1	−1.4 ± 0.3	−2.3 ± 0.4	−3.1 ± 0.7

Data are mean ± SD (*n *=* *9).

* Significantly lower than HYD (*P *<* *0.05).

### Oxygen uptake and whole body substrate use

**Figure 1:**
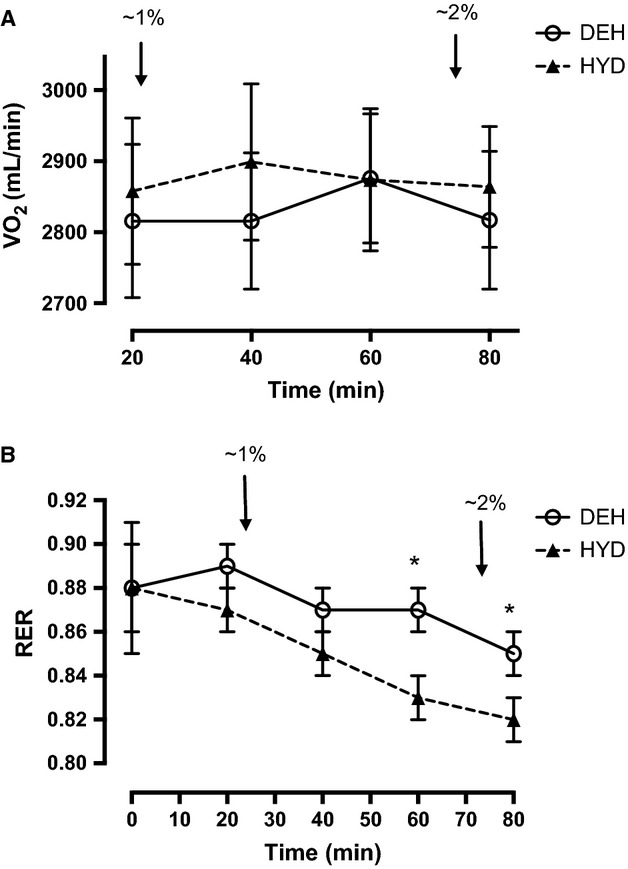
(A) VO_2_ and (B) respiratory exchange ratio (RER) during 90 min of cycling at ∼65% VO_2peak_ in the hydrated (HYD) and dehydrated (DEH) trials. Values are mean ± SD (*n *=* *9). RER was significantly lower than rest at 40, 60, and 80 min in the HYD trial and at 80 min in the DEH trial (*P *<* *0.05). *Significantly greater than HYD trial (*P *<* *0.05). Arrows (↓) indicate ∼1% and ∼2% BM loss.

There was no difference in mean VO_2_ with exercise time and between trials (Fig.1A). The RER progressively decreased in both trials and was lower than rest at 40, 60, and 80 min in the HYD trial and at 80 min in the DEH trial (Fig.1B). RER was higher in the DEH versus HYD trial from 60 to 90 min (Fig.1B).

The rate of CHO oxidation significantly decreased over time in both trials and was lower than 20 min at 60 and 80 min in the HYD trial and at 80 min in the DEH trial. Conversely, the rate of fat oxidation increased over time in both trials and was greater than 20 min at 60 and 80 min in the HYD trial and at 80 min in the DEH trial. There was a trend for CHO oxidation to be greater and fat oxidation to be lower in the DEH trial at all time points, but the difference was not significant until 60 min of cycling. Total CHO oxidation was greater in the DEH trial (HYD, 168 ± 10 g vs. DEH, 202 ± 12 g), and total fat oxidation was lower in the DEH trial (67 ± 1 g vs. 54 ± 1 g).

### Heart rate, core temperature, and rating of perceived exertion

**Figure 2:**
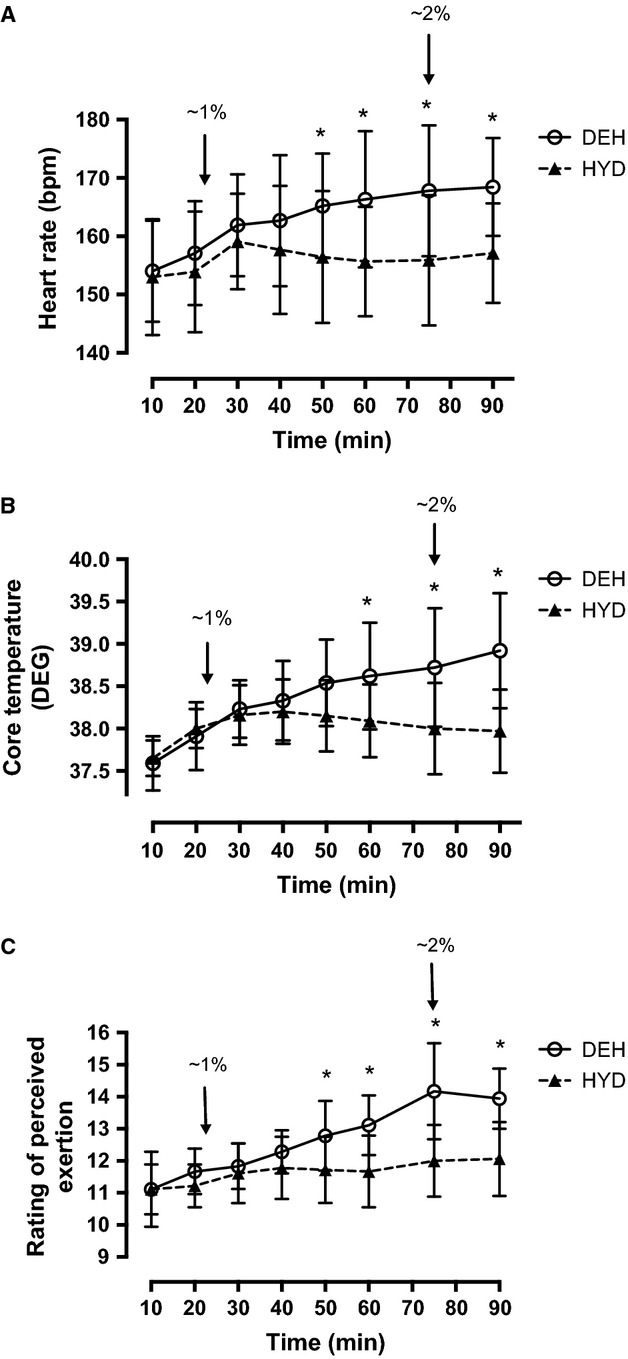
(A) Heart rate response during 90 min of cycling at 65% VO_2peak_ in the hydrated (HYD) and dehydrated (DEH) trials. Heart rate was greater than 10 min at 30–90 min in both trials. (B) Core temperature (°C) during 90 min of cycling at 65% VO_2peak_. (C) Rating of perceived exertion (RPE) during 90 min of cycling at 65% VO_2peak_ in the hydrated (HYD) and dehydrated (DEH) trials. RPE was greater than 10 min at 90 min in the HYD trial and at 40–90 min in the DEH trial. Values are mean ± SD (*n *=* *9). *Significantly greater than HYD trial (*P *<* *0.05). bpm, beats per minute. Arrows (↓) indicate ∼1%, ∼2%, and ∼3% BM loss.

**Figure 3:**
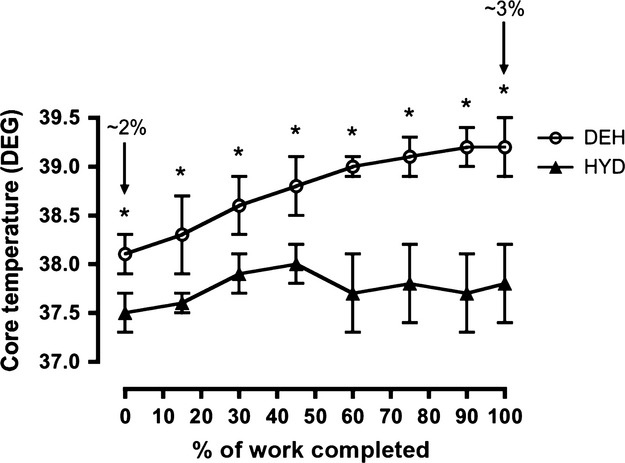
Core temperature (DEG) during the time trial (TT) in the hydrated (HYD) and dehydrated (DEH) trials. Values are mean ± SD (*n *=* *9). *Significantly greater than HYD trial (*P *<* *0.05). Arrows (↓) indicate ∼1%, ∼2%, and ∼3% BM loss.

HR significantly increased over time and was greater than 10 min from 30 to 90 min in both trials (Fig.2A). Subjects had a higher HR from 50 to 90 min of cycling in the DEH versus HYD trial (Fig.2A). During the TT, HR was significantly greater than 15% of work completed at 75–100% of work completed in both trials (Table4). There was no difference in HR between performance trials (Table4). Tc was significantly greater than 10 min at all time points in both trials. Tc was significantly greater in the DEH versus HYD trial from 60 to 90 min (Fig.2B). RPE increased over time in both trials and was greater than 10 min at 90 min in the HYD trial and from 40 to 90 min in the DEH trial (Fig2C). RPE was greater in the DEH trial from 50 to 90 min of cycling (Fig.2C). Throughout the TT, Tc increased only in the DEH trial and was significantly greater than 15% of work completed at 45–100% (Fig.3). Tc was greater in the DEH versus HYD trial at all time points (Fig.3). As well, Tc was greater in the DEH versus HYD trial before the start of the TT (HYD, 37.5 ± 0.2°C vs. DEH, 38.1 ± 0.2°C, Fig.3).

### Blood measurements

Hb and Hct were increased above rest at all time points in both trials (Table2). In the DEH trial, Hb was higher than the HYD trial from 60 min onward (Table2). There was a trend for Hct to be slightly greater in the DEH trial throughout exercise, but the difference was insignificant (Table2). Pvol loss was greater than rest at all time points in both trials and was greater in the DEH versus HYD trial from 75 min onward (Table2). Posm was greater than rest at 60–90 min in the HYD trial and from 40 to 90 min in the DEH trial (Table2). Posm was greater in the DEH versus HYD trial from 40 min onward (Table2). During the TT, Hb, Pvol loss, and Posm were significantly greater at TT1 and TT2 in the DEH versus HYD trial. Blood glucose decreased with exercise time and was lower than rest at 40–90 min in the HYD trial and at 90 min in the DEH trial, with no differences between trials (Table2). Blood lactate (La) was increased from rest at 20 min of exercise and beyond in both trials and was higher in the DEH versus HYD trial at 40–90 min (Table2). As well, during the TT DEH subjects had a blood [La] that was the same as the HYD subjects at TT1 and a significantly higher blood [La] at TT2 despite power output being significantly lower (Table2). Plasma FFA was not affected with exercise time and there was no difference between trials. Plasma EPI significantly increased from rest at all time points in both trials, however, there were no differences between trials at any time point (Table2). During the TT, plasma EPI was greater in both trials at TT2 compared to TT1, with no between trial differences (Table2). During the 90-min trial there was no effect of exercise time or trial differences in serum Hsp72 (Table2). Serum Hsp72 content was significantly greater than rest at TT1 and TT2 in both trials, and was significantly greater at both time points of the TT in the DEH versus HYD trial (Table2).

**Table 2:** Hemoglobin (Hb), hematocrit (Hct), glucose, lactate, and plasma volume loss (Pvol loss), plasma osmolality (Posm), plasma free fatty acid (FFA), plasma epinephrine (EPI) concentration, and serum heat shock protein (Hsp) 72 content during 90 min of cycling at ∼65% VO_2peak_ and at 50% (TT1) and 90% (TT2) of work completed in the time trial in the hydrated (HYD) and dehydrated (DEH) trials

	Trial	Time (min)						Time trial	
		0	20	40	60	75	90	TT1	TT2
Hb	HYD	13.9 ± 0.4	14.5 ± 0.4*	14.5 ± 0.4*	14.6 ± 0.4*	14.7 ± 0.3*	14.6 ± 0.2*	14.7 ± 0.3*	14.8 ± 0.4*
(g/100 m)	DEH	13.9 ± 0.5	14.8 ± 0.5*	14.8 ± 0.5*	15.0 ± 0.3†*	15.2 ± 0.5†*	15.3 ± 0.4†*	15.5 ± 0.4†*	15.7 ± 0.3†*
Hct	HYD	41.1 ± 0.8	42.2 ± 0.8†	42.5 ± 0.7*	42.2 ± 0.7*	42.6 ± 0.7*	42.7 ± 0.6*	43.0 ± 0.6*	42.9 ± 0.6*
(%)	DEH	41.6 ± 0.8	42.7 ± 0.7*	42.6 ± 0.7*	42.5 ± 0.7*	42.8 ± 0.7*	42.9 ± 0.6*	43.5 ± 0.6*	43.7 ± 0.6*
Pvol loss	HYD	–	−3.6 ± 0.7*	−3.4 ± 0.9*	−3.9 ± 0.9*	−3.8 ± 1.2*	−4.9 ± 1.3*	−5.0 ± 0.9*	−5.2 ± 1.2*
(%)	DEH	–	−3.8 ± 0.9*	−4.1 ± 0.9*	−4.7 ± 1.4*	−5.3 ± 1.7*†	−7.7 ± 2.4*†	−8.0 ± 1.5*†	−11.4 ± 5.7*†‡
Posm	HYD	288 ± 1.5	290 ± 1.5	288 ± 1.1	287 ± 1.8*	286 ± 1.6*	286 ± 1.9*	290 ± 1.7	293 ± 1.6*
(mOsmol/kg^−1^)	DEH	290 ± 1.1	292 ± 1.0*	293 ± 1.2*†	293 ± 1.2*†	294 ± 0.9*†	296 ± 0.9*†	301 ± 1.0*†	300 ± 0.6*†
Glucose	HYD	3.9 ± 0.3	3.9 ± 0.2	3.6 ± 0.1*	3.6 ± 0.2*	3.7 ± 0.1*	3.7 ± 0.1*	3.8 ± 0.2*	3.7 ± 0.1*
(mmol/L)	DEH	3.9 ± 0.1	4.0 ± 0.1	3.9 ± 0.1	3.9 ± 0.1	4.0 ± 0.1	3.7 ± 0.2*	3.8 ± 0.1*	3.8 ± 0.2*
Lactate	HYD	0.9 ± 0.1	2.6 ± 0.4*	2.5 ± 0.4*	1.7 ± 0.3*	2.0 ± 0.3*	1.9 ± 0.3*	8.0 ± 1.0*	6.1 ± 1.4*
(mmol/L)	DEH	0.9 ± 0.4	2.5 ± 0.4*	3.1 ± 0.5*†	2.6 ± 0.4*†	2.5 ± 0.4*†	2.6 ± 0.4*†	7.8 ± 1.1*	8.0 ± 0.8*†
Plasma FFA	HYD	0.7 ± 0.03	–	–	0.7 ± 0.04	–	0.7 ± 0.1	0.6 ± 0.1	0.6 ± 0.1
(mmol/L)	DEH	0.7 ± 0.04	–	–	0.8 ± 0.03	–	0.8 ± 0.1	0.7 ± 0.1	0.7 ± 0.1
Plasma EPI	HYD	0.8 ± 0.1	–	–	–	–	1.1 ± 0.1*	1.4 ± 0.2*	2.1 ± 0.3*‡
(nmol/L)	DEH	0.7 ± 0.1	–	–	–	–	1.2 ± 0.1*	1.6 ± 0.1*	2.5 ± 0.3*‡
Serum Hsp72	HYD	0.8 ± 0.2	0.9 ± 0.2	–	0.9 ± 0.2	–	0.9 ± 0.2	1.2 ± 0.3*	1.3 ± 0.2*
(ng/mL)	DEH	0.8 ± 0.1	0.9 ± 0.2	–	1.0 ± 0.3	–	1.1 ± 0.3	1.4 ± 0.4*†	1.6 ± 0.3*†

Data are mean ± SD (*n *=* *9).

* Significantly greater than 0 min (*P *<* *0.05).

† Significantly greater than HYD (*P *<* *0.05).

‡ Significantly greater than TT 1 (*P *<* *0.05).

### Muscle fuels and metabolites

**Figure 4:**
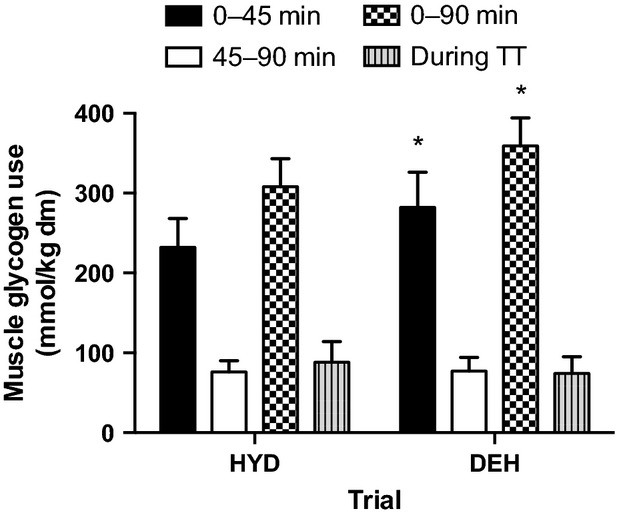
Muscle glycogen use from 0 to 45 min, 45 to 90 min, 0 to 90 min, and during the time trial (TT) in the hydrated (HYD) and dehydrated (DEH) trials. Values are mean ± SD (*n *=* *9). *Significantly greater than HYD trial (*P *<* *0.05).

Muscle PCr was lower than rest at all time points in both trials, but not different between trials and [Cr] was reciprocal with the PCr changes (Table3). Muscle ATP content was not altered with exercise in the HYD trial but was lower than rest after the TT in the DEH trial with no difference between trials (Table3). ADPf and AMPf were higher than rest at all time points during exercise in both trials, with no differences between trials (Table3). Muscle La was greater than rest at all time points in both trials and greater in the DEH trial at 90 min and after the TT (Table3). Muscle glycogen content was similar in the two trials before exercise and significantly lower after 45 and 90 min, and after the TT in both trials compared to rest (Table3). In the DEH trial, there was more glycogen used in the first 45-min (15%, HYD, 232 ± 36 vs. DEH, 282 ± 44 mmol/kg dm, *P *=* *0.05) and over the entire 90-min trial (17%, HYD 308 ± 35 vs. DEH, 359 ± 35 mmol/kg dm, *P *=* *0.04), with a trend for greater glycogen use from 45 to 90 min (24%, HYD, 76 ± 14 vs. DEH, 77 ± 17 mmol/kg dm, *P *=* *0.29) (Fig.4). There was no difference in muscle glycogen use between trials during the TT (HYD, 88 ± 26 vs. DEH, 74 ± 21 mmol/kg dm).

**Table 3:** Skeletal muscle fuel and metabolite contents during 90 min of cycling at ∼65% VO_2peak_ and immediately after the completion of the time trial (TT) in the hydrated (HYD) and dehydrated (DEH) trials

Time	HYD				DEH			
	0	45	90	Post-TT	0	45	90	Post-TT
PCr	90.9 ± 5.4	72.1 ± 5.6*	68.4 ± 5.1*	62.3 ± 4.2*	88.1 ± 5.5	71.1 ± 4.2*	69.9 ± 6.0*	65.2 ± 5.1*
Cr	71.1 ± 5.5	90.0 ± 5.7*	93.6 ± 5.0*	99.7 ± 4.8*	74.0 ± 3.3	90.9 ± 4.2*	92.1 ± 5.6*	96.8 ± 6.6*
ATP	28.6 ± 3.6	23.5 ± 1.6	24.4 ± 1.6	24.3 ± 1.9	28.2 ± 2.3	23.5 ± 2.3	23.5 ± 2.1	22.2 ± 1.6*
ADPf (*μ*mol/kg dm)	124 ± 14	184 ± 40*	199 ± 24*	197 ± 20*	137 ± 13	167 ± 9*	190 ± 30*	195 ± 42*
AMPf	0.6 ± 0.1	1.7 ± 0.8*	1.7 ± 0.4*	1.7 ± 0.3*	0.7 ± 0.1	1.2 ± 0.1*	1.6 ± 0.4*	1.9 ± 0.7*
(*μ*mol/kg dm)								
Lactate	0.7 ± 0.2	2.2 ± 0.4*	1.9 ± 0.3*	7.0 ± 1.3*	0.6 ± 0.2	2.4 ± 0.6*	2.3 ± 0.5*†	8.2 ± 1.5*†
Glycogen	467 ± 26	221 ± 25*	159 ± 22*	72 ± 19*	499 ± 39	217 ± 25*	140 ± 24*	66 ± 13*

Data are mean ± SD (*n *=* *9), mmol/kg dry muscle (dm).

PCr, phoshocreatine; Cr, creatine; ATP, adenosine triphosphate; ADPf, free adenosine diphosphate; AMPf, free adenosine monophosphate.

* Significantly greater than 0 min (*P *<* *0.05).

† Significantly higher than HYD trial (*P *<* *0.05).

### Hsp72 protein changes

**Figure 5:**
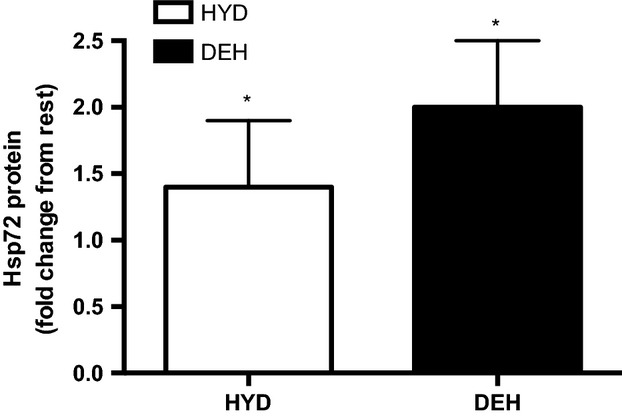
Skeletal muscle heat shock protein (Hsp) 72 protein change at rest and at the end of the time trial (TT) in the hydrated (HYD) and dehydrated (DEH) trials. Values are mean ± SD (*n *=* *9). *Significantly greater than rest (*P *<* *0.05).

There was no difference between trials in Hsp72 protein content at rest. Skeletal muscle Hsp72 protein was significantly greater than rest at the end of the TT in both the HYD (1.4-fold increase) and DEH (2.0-fold increase) trials. There was no significant difference between trials (Fig.5).

### Time trial performance

Subjects in the DEH trial were 13% slower in completing the TT than the HYD trial (HYD 31.8 ± 4.1 vs. DEH 36.0 ± 3.1 min, Table4). Power output was significantly lower in the DEH versus HYD trial throughout the TT (Table4).

**Table 4:** Performance time, heart rate, and power output at 15%, 30%, 45%, 60%, 75%, 90%, and 100% of work completed during the time trial in the hydrated (HYD) and dehydrated (DEH) trials

Work completed	15%	30%	45%	60%	75%	90%	100%
Time (min)							
HYD	–	–	–	–	–	–	31.8 ± 4.1
DEH	–	–	–	–	–	–	36.0 ± 3.1*
Heart rate (bpm)							
HYD	180 ± 3	182 ± 3	182 ± 3	183 ± 3	184 ± 3†	185 ± 3†	187 ± 3†
DEH	179 ± 2	181 ± 2	180 ± 3	179 ± 4	183 ± 3†	183 ± 2†	188 ± 3†
Power output (Watts)							
HYD	252 ± 18	239 ± 17	229 ± 16	224 ± 16	228 ± 15	229 ± 22	266 ± 19
DEH	220 ± 16‡	208 ± 14‡	203 ± 16‡	204 ± 16‡	217 ± 15‡	231 ± 20‡	250 ± 19‡

Data are mean ± SD (*n *=* *9).

* Significantly slower than HYD (*P *<* *0.05).

† Significantly greater than 15% of work completed (*P *<* *0.05).

‡ Significantly less than HYD (*P *<* *0.05).

## Discussion

This study investigated the effects of starting exercise mildly dehydrated combined with exercise-induced progressive dehydration on whole body substrate oxidation, skeletal muscle metabolism, serum Hsp72 response, and TT performance. All physiological and blood parameters, in addition to carbohydrate oxidation and muscle glycogen use, were greater during the 90-min trial when subjects were dehydrated by ∼1–2% BM. TT performance was significantly compromised (13%) with dehydration of ∼2–3% BM loss, as power output was lower throughout the TT. Additionally, DEH subjects had higher Tc, Posm, blood and muscle lactate, and serum Hsp72 levels. Muscle glycogen content was already very low at the start of the TT in both trials and may have limited the performance in both the DEH and HYD trials.

### Effect of dehydration on substrate oxidation and muscle glycogen use

This study demonstrated that whole body CHO oxidation was greater when subjects were dehydrated by ∼1.5% BM. There was a trend for CHO oxidation to be greater in the DEH trial throughout exercise even at the beginning of the trial when subjects were 0.6% dehydrated from the overnight fluid restriction, however, the difference between trials was not significant until 60 min of exercise when subjects had lost ∼1.5% BM (1% = ∼22.5 min; 2% = 75 min, and 3% = post TT). There are few studies that have demonstrated an effect of mild dehydration on RER in a temperate environment. Fallowfield et al. (Fallowfield et al. 1996) demonstrated that RER significantly increased with progressive dehydration to 2.7% BM loss. In their study, male subjects ran on a treadmill at 70% VO_2peak_ until exhaustion with no fluid (NF) or with fluid replacement (FR), consuming 2 mL/kg BM every 15 min throughout exercise. CHO oxidation was significantly increased (NF 73% vs. FR 64% of total energy expenditure) and fat metabolism was suppressed after 75 min of exercise in the NF trial compared with the FR trial (0.90 vs. 0.86) when dehydration amounted to 2.7% BM loss. Hargreaves et al. (Hargreaves et al. 1996) reported a higher RER in males after 60 and 120 min of cycling at 65% VO_2peak_ with progressive dehydration to 2.9% BM loss versus staying euhydrated. One novel aspect of the present study is that changes in substrate utilization (increased CHO oxidation) were already present at mild levels of dehydration (∼1–2%).

In addition, total muscle glycogen use was greater during the 90-min trial when subjects were 1–2% dehydrated. Hargreaves et al. (Hargreaves et al. 1996) demonstrated an increased reliance on muscle glycogen, greater muscle lactate, and higher rectal and muscle temperatures after 2 h of moderate intensity exercise-induced dehydration to 2.9% BM loss. More recently, we reported a 24% increase in total muscle glycogen use over 2 h of cycling at 65% VO_2peak_ in male subjects dehydrated to 2.7% BM loss, with no difference in plasma [EPI] or the energy status of the muscle, but a greater Tc between trials (Logan-Sprenger et al. 2013a). There appears to be at least three potential mechanisms that may account for the upregulated glycogen phosphorylase (PHOS) activity and subsequent glycogen use with dehydration. They include an increased sympathoadrenal response leading to elevated circulating [EPI] and activation of PHOS, a decreased energy status in the cell manifested by elevated ADPf and AMPf levels, and increased muscle temperature (Tm) (Febbraio 2000). In the present study, there were no differences in plasma [EPI] or the energy status of the cell, which is consistent with the findings of Logan-Sprenger et al. (Logan-Sprenger et al. 2013a, 2013b). However, Tc was significantly greater in DEH subjects with 1–2% BM loss. In light of this, we suggest that an increased Tm effect is the most plausible mechanism to explain the increased muscle glycogenolysis during progressive dehydration in males.

### Effect of dehydration on Hsp72 response

Heat shock proteins (Hsps) are a group of highly conserved proteins present in all cells and expressed in low concentrations in the basal state. The Hsp70-kDa family is considered to be the most highly inducible form of Hsp and is stimulated by a variety of pathological, physiological, and environmental stressors. The only known measureable form of the Hsp70-kda family in the skeletal muscle is Hsp72, functioning in cytoprotection mediating antiapoptosis, antioxidative activity, neuroprotection, immunoprotection, and as a molecular chaperone (Horowitz and Robinson 2007). Exercise has been shown to increase Hsp72 gene and protein in serum and contracting skeletal muscle following acute exercise (Febbraio et al. 2002), but the effect of hydration alone on Hsp72 response has not been previously investigated. We measured serum and muscle Hsp72 to determine if dehydration and exercise posed a greater systemic and cellular stress than exercise alone. We demonstrated that serum Hsp72 was greater during the performance trial with ∼2–3% BM loss indicating greater systemic stress with mild dehydration at high-intensity exercise. Krause and Rodrigues-Krause (Krause and Rodrigues-Krause 2011) suggested that moto neurons are very sensitive to heat and potentially one of the most stressed cells during exercise and may be the major site for the extracellular Hsp72 function. They proposed that extracellular Hsp72 during exercise can be taken up by the moto neuron and act as an intracellular chaperone yielding cytoprotection against oxidative damage, heat, and protein denaturation, along with many other stressors to help prevent neurodegeneration (Krause and Rodrigues-Krause 2011). Although this study did not determine what tissue the serum Hsp72 was being released from, it has been speculated by Febrraio et al. (Febbraio et al. 2002) that the splanchnic tissues release Hsp72 during exercise and are partly responsible for the elevated serum Hsp72 content.

Additionally, there was a trend for greater muscle Hsp72 protein after the TT in DEH versus HYD subjects suggesting that the muscle cell is under greater stress when dehydrated by ∼2–3% BM. Febbraio et al. (Febbraio et al. 2002) suggested that intact muscle cells are not able to release Hsp72 into the circulation, but that stressed muscle cells synthesize Hsp72 in order to protect intracellular proteins from unfolding and denaturation and also exert an anti-inflammatory effect. The consequences of increasing muscle Hsp72 are currently unknown.

### Effect of dehydration on TT performance

The results of this study revealed that TT performance was significantly compromised (13%) when subjects were 2.3–3.1% dehydrated versus maintaining BM. In the DEH trial, HR, Tc, Posm, and blood and muscle lactate were all significantly higher despite subjects cycling at a lower mean power output compared to the HYD trial. These results are physiologically meaningful and practical as Ebert et al. (Ebert et al. 2007) reported that male and female elite road cyclists during the Tour de France and the Tour de l’Aude cycling tours lost on average 2.8% and 2.6% BM loss per stage over the 21- and 10-day tours, respectively (Ebert et al. 2007). Similar to our findings, Bardis et al. (Bardis et al. 2013) reported that well-trained male endurance cyclists completed a 5-km hill climb 5.8% faster when dehydration was minimized (1.4% BM loss) versus greater dehydration (2.2% BM loss). They also reported that the more dehydrated athletes had a significantly higher Tc and RPE upon finishing the race. In light of our results and the current literature, it appears that high-intensity performance cannot be sustained when dehydration amounts to ∼2–3% BM loss.

Cycling for 90 min at 65% VO_2peak_ depleted much of the muscle glycogen in the subjects, leaving little for use during the TT. The TT began in both trials with low and similar muscle glycogen contents (HYD, 159 ± 22 vs. DEH, 140 ± 24 mmol/kg dm) and there was no difference in muscle glycogen use between trials during the TT (HYD, 88 ± 26 vs. DEH, 74 ± 21 mmol/kg dm). Therefore, the results suggest that greater muscle glycogen use was not the reason for the decreased TT performance when dehydrated in the present exercise protocol, as there was little to use in either trial. Instead we suggest that a critically high Tc is the more domineering factor determining performance when dehydrated by 2–3% BM. There are multiple studies suggesting the presence of anticipatory pacing, with the specific goal being to ensure that a thermoregulatory failure does not occur during demanding exercise; thus, behavior modification guarantees that homeostasis is protected under all conditions (Tatterson et al. 2000; Marino 2004; Tucker et al. 2004, 2006). In this study the exacerbated physiological parameters (increased Tc, HR, Posm, serum Hsp72, and decreased Pvol) during the DEH performance trial provided the physiological feedback, which may have led to the reduction in self-selected pace in an attempt to prevent the early onset of fatigue. The extra feedback in the DEH trial may have been responsible for the increased RPE, decreased motivation, and the slowing of self-selected pace during high-intensity endurance exercise when dehydrated by ∼2–3% BM loss.

## Conclusions

This study demonstrated that many physiological parameters along with CHO oxidation and muscle glycogen use were greater during 90 min of moderate intensity exercise when subjects progressively dehydrated from 0.6% to 2.3% of BM versus maintaining BM through drinking. Subsequent TT performance was 13% slower when subjects began 2.3% and ended 3.1% dehydrated. Throughout the TT, Tc, Posm, blood and muscle lactate, and serum Hsp72 were higher, even while working at a lower power output. Differences in muscle glycogenolysis with dehydration did not explain the decrease in TT performance as subjects began the performance trials with similar and low muscle glycogen contents in the DEH and HYD trials. Instead, the higher physiological variables and the higher Tc may account for the performance detriments when dehydrated as the body reduces the exercise intensity to prevent further heat production.

The practical application of this study demonstrated that athletes training in a dehydrated state induce a greater cellular and whole body stress, which in turn may elicit an enhanced training adaptation. However, this greater cellular and whole body stress including an elevated core temperature significantly decreases performance and attention needs to be paid to hydration status and cooling strategies during competitions.

## Conflict of Interest

None declared.
